# Diverse Effect of Two Cytokinins, Kinetin and Benzyladenine, on Plant Development, Biotic Stress Tolerance, and Gene Expression

**DOI:** 10.3390/life11121404

**Published:** 2021-12-15

**Authors:** Zoltán Bozsó, Balázs Barna

**Affiliations:** Centre for Agricultural Research, ELKH, Plant Protection Institute, Herman Ottó Str. 15, H-1022 Budapest, Hungary; bozso.zoltan@atk.hu

**Keywords:** benzyladenine, kinetin, *Arabidopsis*, tobacco, *Tobacco mosaic virus*, *Pseudomonas syringae*, *Botrytis cinerea*, gene expression changes, chlorophyll content

## Abstract

The plant hormones cytokinins affect a various array of plant growth and development processes as well as responses to biotic and abiotic stresses. In this study, the opposite effect of two different cytokinins kinetin (N^6^-furfuryladenine) and benzyladenine (BA) on development and on the tolerance of *Arabidopsis* and tobacco plants to virus, bacteria, and fungi infection was reported. Treatments of *Arabidopsis* and tobacco seedlings with saturated solutions of BA inhibited plant progress, while treatments with saturated water solution of kinetin promoted plant development. Furthermore, BA pre-treatments strongly reduced the number of TMV (*Tobacco mosaic virus*) lesions on tobacco and the tissue damage caused by the incompatible *Pseudomonas* bacteria on *Arabidopsis* and tobacco leaves. Similarly, BA pre-treatment significantly reduced the necrotic disease symptoms of *Botrytis cinerea* infection. Kinetin pre-treatments had a much weaker or no protective effect on the damage caused by the above pathogens. Accordingly, *Arabidopsis* gene expression profiles after treatments also showed that the two cytokinins have different effects on several plant processes. The gene expression results supported the more robust effect of BA, which up and downregulated more than 2000 genes, while only 436 genes were influenced by kinetin treatment. It is noteworthy that BA and kinetin treatment changed gene expressions in the same direction only in a relatively few cases (73 upregulated and 70 downregulated genes), and even 28 genes were regulated into the opposite directions by BA and kinetin. Both treatments had a strong effect on auxin and gibberellin-related genes, but only BA had a significant effect on cytokinin-induced processes. While kinetin exclusively activated the flavonoid synthesis genes, BA affected more significantly protein synthesis, photosynthesis, and plant defence-related genes. In conclusion, BA solution had sometimes the opposite and generally a much stronger effect than kinetin solution not only on the development and on biotic stress tolerance of tobacco and *Arabidopsis* plants but also on the gene expressions. The stronger protective effect of BA to necrotic stresses is probably due to its stronger senescence inhibitory effect on plant tissues, as supported by the stronger chlorophyll retardation of the BA-treated leaves.

## 1. Introduction

Although much is known about cytokinins, still, many important open questions remain. Since in preliminary experiments, we found that kinetin and benzyladenine pre-treatments have different effects on the development of *Arabidopsis* plants, our aim was to compare the effect of these two cytokinins on *Arabidopsis* and tobacco plants under the same conditions.

Cytokinins are a class of plant hormones that promote cell division, plant growth, and plant development, in addition to responses to abiotic and biotic stresses. Moreover, cytokinins have been shown to slow the senescence of plants and assembling nutrients from nearby tissues [1,2,3,4]. Furthermore, cytokinins are signalling molecules acting both locally and at a distance [5,6]. The first cytokinin to be identified was kinetin as a degradation product of DNA that promotes plant cell division. Evidence for the presence of kinetin in natural products has been provided by mass spectrometric analysis of DNA components [7]. Both benzyladenine and kinetin are adenine-type cytokinins with a similar chemical structure, but in BA, a benzyl group can be found instead of the furfuryl group. It is noteworthy that senescence was delayed by kinetin and BA in an oat leaf test, but not by natural cytokinins, zeatin, and isopentenyl adenine [8]. In addition, both benzyladenine and kinetin show antioxidant properties in human skin fibroblast and in mammalian cells [9,10].

Elevated cytokinin and auxin content has a pivotal role in keeping juvenility and the active metabolism of plant tissue infected by biotrophs, such as rust or powdery mildew (green island syndrome), and in directing nutrient transport to the infected plant parts. On the other hand, it has been known for a long time that the senescence or juvenility of plant tissues has a strong effect on their reactions to abiotic stresses and pathogen attacks [11]. Generally, necrotrophic pathogens prefer senescent tissues, but biotrophic pathogens prefer juvenile tissues, while hemibiotrophic pathogens such as *Pseudomonas* bacteria or *Phytothora infestans* are in some respect in between [12,13]. Accordingly, any type of inhibition of plant senescence, such as treatment with cytokinins or transformation with cytokinin biosynthesis genes shall improve the resistance of plants to necrosis-inducing necrotrophic pathogens such as *Botrytis cinerea* or *Sclerotinia* ssp., but it shall increase susceptibility to biotrophic pathogens such as powdery mildew or rust [14]. In addition, many studies demonstrate that a number of senescence-associated transcription factors (Sen-TFs) function as positive or negative regulators of plant immunity [11,13].

Although in the past decades, significant progress has been made in the understanding of the molecular mechanisms of plant disease resistance, still, there are many open questions [11,15,16,17]. This statement is also valid regarding the role of plant hormones and especially of cytokinins in plant immunity [18,19,20]. In *Arabidopsis*, it was suggested that cytokinin-mediated resistance functions through SA-dependent mechanisms based on the finding that ARR2 (a positive regulator of cytokinin signalling) interacts with TGA3 (a transcription factor involved in inducing SA-responsive genes) in the regulation of the disease marker gene PR1 against biotrophic infections in plants [18]. Furthermore, cytokinin overexpressing transgenic plants and exogenous cytokinins feeding approaches unanimously resulted in “more cytokinins less disease symptoms” and vice versa [21]. Similarly, we also found that cytokinin overproduction induced the juvenility of plant tissues correlated with augmented antioxidant activities, and it is connected to elevated tolerance to necrotic symptoms caused by pathogens [14,22,23,24].

Here, we report on the diverse, sometimes opposite effect of the two cytokinins, kinetin and benzyladenine, on *Arabidopsis* and tobacco plant development, chlorophyll content of senescent leaves, and their tolerance to virus, bacteria, and fungi, in addition to the effect on gene expressions.

## 2. Materials and Methods

### 2.1. Plant Materials and Pathogens

Experiments were carried out with the wild-type Columbia ecotype of *Arabidopsis thaliana* (L.) Heynh, and *Nicotiana tabacum* L. cv. Xanthi-nc carrying the hypersensitive *N* resistance gene to TMV. Seeds of *Arabidopsis* plants were sown into pots and kept 2 days at 5 °C in dark; then, they were put into a growth chamber for an 11 h photoperiod light/13 h dark, 140 µmol m^−2^ s^−1^ fluorescent lightening. Tobacco plants were grown under standard greenhouse conditions (18–23 °C; about 16 h daylight with 160 µmol m^−2^ s^−1^ supplemental light for 8 h per day; relative humidity: 75–80%).

Tobacco leaves were inoculated with a suspension of the U1 strain of *Tobacco mosaic virus* (TMV) as described earlier [25]. Briefly, the virus was maintained in *N. tabacum* cv. Samsun plants carrying no *N* resistance gene, and leaves showing typical symptoms of TMV infection were ground (1 g in 10 mL of 10 mM sodium phosphate buffer, pH 7.0) in a mortar, and the homogenate was used for inoculation.

Two strains of the hemibiotrophic *Pseudomonas* bacteria were used: *P. syringae* pv. *syringae* 61 (*P.s*. pv. *s.* 61 [26]) and *P. syringae* pv. *tomato* strain DC3000 (*P.s.* pv. *t.* DC3000 [27]). *P.s*. pv. *s.* 61 elicits the hypersensitive response (HR) in non-host plants. *P.s.* pv. *t.* DC3000 is the cause of bacterial speck disease on tomato and *Arabidopsis*, and it gives a compatible reaction on Columbia ecotype. Bacterial cultures were maintained on nutrient agar at 30 °C. Cultures were transferred to fresh medium 16 to 24 h prior to use. For infection, all fully developed plant leaves were brushed either with water (control) or with bacterial suspension (10^8^ cfu cm^−3^) [28]. In the case of tobacco, in addition to incompatible *P. s.* pv. *s.* 61, suspensions of the compatible *P.s*. pv. *tabaci* (*P.s.* pv. *tabaci, NCAIM1 B.01601-National Collection of Agricultural and Industrial Microorganisms, Budapest, Hungary*) bacteria were injected into the leaves and evaluated by scoring (from 0 to 4) the necrotic damage caused by bacterial suspensions injected into tobacco leaves 3 days post inoculation.

As a necrotrophic pathogen, *Botrytis cinerea* strain b05.10 [29] was used. Infection was carried out on leaves derived from 50–55-day-old tobacco plants treated with 10 mL water, BA, or kinetin solutions for 14 days. The leaves were placed on wet filter papers in glass Petri dishes (28 cm in diameter) and inoculated with 0.5 diameter agar discs from *Botrytis cinerea* 5-day-old culture. Evaluation of the symptoms was carried out by measuring lesion development on the leaves. The significance of the difference between two values of disease damage was evaluated by *t*-test.

### 2.2. Experimental Design and Chlorophyll Content Determination

Saturated water solutions of kinetin (0.349 mM) or BA (0.266 mM) were used for treatments throughout the experiments. Older and younger *Arabidopsis* and tobacco plants were used to examine the effect of the two cytokinins on plants of different ages. For *Arabidopsis* treatments, either plant leaves that were 45–50 days old were brushed with the solutions, or *Arabidopsis* plants that were 30–35 days old were sprinkled with 5 mL solution to the root every day for 14 days. In the case of tobacco at an early seedling stage (35–40 days old) or at a later stage (50–55 days old), plants were sprinkled with 5 or 10 mL solution respectively to the root for 10 days. Water-treated plants were used as controls.

In separate experiments, Xanthi tobacco plants that were 50–55 days old were sprinkled with saturated BA, saturated Kin, or water solutions in 3 groups. The first group of plants received 30 mL of cytokinin solutions only on the first day, the second group received theirs three days after the first doses had the second 30 mL (altogether 60 mL) of cytokinin solutions, and the third group of plants again after 3 days received a third 30 mL (altogether 90 mL) of test solutions.

Chlorophyll content was measured in the second fully developed leaves (from the soil level) of tobacco plants. Five weeks after the last treatment with the cytokinin solutions, chlorophyll was extracted with 80% acetone from tobacco leaves and determined at 647 and 660 nm as described earlier [30].

### 2.3. RNA Purification

For one RNA sample, approximately 100 mg of leaf tissues were collected from three BA, kinetin, or water-treated plants at indicated times. The samples were frozen in liquid nitrogen and stored at −70 °C. To obtain three independent biological replicates, sample collections were repeated with three plant generations. Total RNA was extracted from frozen tissues using Qiagen RNeasy Plant Mini kit and Qiagen Rnase-free Dnase Set (Qiagen, Germantown, MD, USA). RNA purity was determined through a Nanodrop ND-1000 (NanoDrop Technologies Inc., Wilmington, DE, USA), and the integrity was checked by 2100 Bioanalyser (Agilent Technologies Inc., Santa Clara, CA, USA).

### 2.4. Microarray

Agilent Arabidopsis (V4) Gene Expression Microarray, 4 × 44K Array (one glass slide formatted with four high-definition 44K arrays) were used for gene expression detections.

Amplification and labelling were performed according to the Agilent protocol (Two-Color Microarray-Based Gene Expression Analysis Version 6.7). The method uses a T7 RNA Polymerase Blend, which simultaneously amplifies the target material and incorporates Cyanine 3-CTP or Cyanine 5-CTP.

The hybridization protocol was processed through a hybridization oven (Agilent Technologies Inc., Santa Clara, CA, USA). Then, a gasket slide (Agilent Technologies Inc., Santa Clara, CA, USA) was stuck on the microarray slide and then put it in to Agilent Microarray Hybridization Chamber (Agilent Technologies Inc., Santa Clara, CA, USA). The hybridization master mix solution was prepared with components from a Gene Expression Hybridization Kit (Agilent Technologies Inc., Santa Clara, CA, USA) and prepared according to the Agilent protocol. Hybridization solution samples were mixed and loaded onto the gasket slide surface. Then, the microarray slide was added on the top of the gasket slide. Three assembled slides chambers were placed in a rotisserie in a hybridization oven, and they were hybridized for 17 h at 65 °C. The hybridization rotator was set to rotate at 10× *g*. Post-hybridization washing was done using Gene Expression Wash Buffer Kits with increasing stringency (Agilent Technologies Inc., Santa Clara, CA, USA). Microarrays were scanned with InnoScan 900 (Innopsys, Carbonne, France) at a resolution of 5 μm. Grid files (gpr file) were created in Mapix software (Innopsys, Carbonne, France) through a microarray image file and a microarray layout file (.gal file). All data processing was performed using functions from package limma in R. We performed background correction by the method “normexp” [31] at first. As a second step, we performed within array normalization by the method “loess” [32,33,34], and then, we performed between array normalization by method “Aquantile” [35]. As a last step of data preprocessing, we replaced all groups of replicated probes by their average value. The differential expression was also computed with use of package limma, particularly by functions lmFit, eBayes, and top Table.

### 2.5. Primer Design and Quantitative RT-PCR Analysis of Gene Expression

DNase-treated (TURBO DNA-free Kit, Thermofisher, Waltham, MA, USA) total RNA (1.5 µg) was used for the synthesis of 20 μL cDNA (High-Capacity cDNA Reverse Transcription Kit, Applied Biosystems) with random primers. Two and a half µL from a 10-fold dilution of cDNA stock was used in each 15 µL reaction using a qPCRBIO SyGreen Mix Lo-ROX (PCR Biosystems, London, UK) real-time PCR mix. The final primer concentrations in 15 µL PCR reaction were 0.3 µM. Real-time PCR amplifications were performed in a DNA Engine Opticon 2 thermocycler (MJ Research, Hercules, CA, USA). The cycling parameters were 95 °C for 3 min followed by 40 cycles of 95 °C for 10 s and 60 °C for 30 s. Melting curve runs were also performed at the end of each PCR reaction to verify the presence of a single product. Measured C(T) values were normalized to the constitutive expressed ubiquitin-conjugating enzyme AT1G14400 C(T) values (forward primer: CTCTGTGACCCTAATCCGAATTCT; reverse primer: GCGCTTGCTTTCGCTGTAC). A total of 24 representative genes that showed significant expression changes in microarray experiments were used for qPCR validating measurements (Supplementary Table S2). The relative quantification of gene expression was carried out using the comparative cycle threshold [C(T)] method for the calculation of ΔC(T) and ΔΔC(T) values. Three biological replicates of treated (benzyladenine or kinetin) and untreated control samples were used for PCR, and the averages of the treated values were divided by the values of untreated values.

## 3. Results

### 3.1. Benzyladenine and Kinetin Have Different Effects on Plant Development

**Figure 1:**
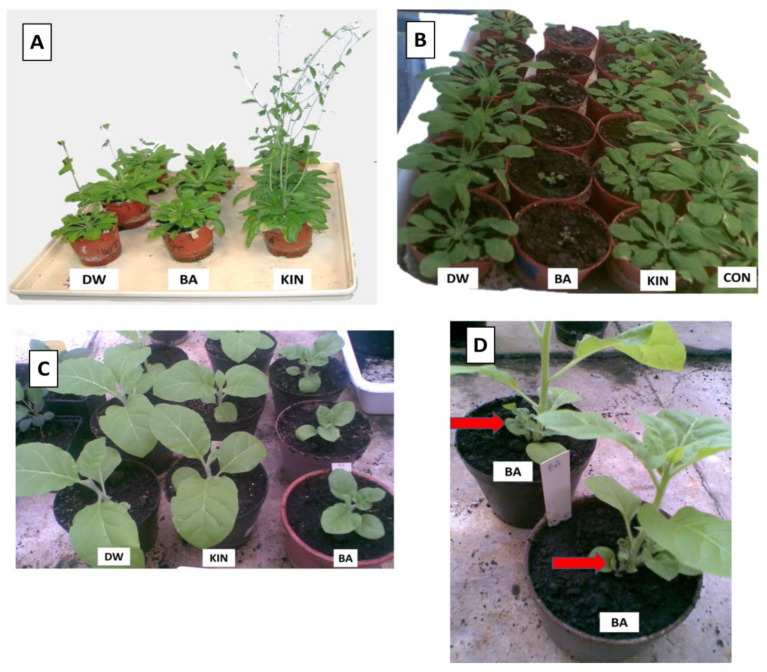
Effect of distilled water (DW), benzyladenine (BA), or kinetin (KIN) solutions on *Arabidopsis* (**A**,**B**) and tobacco (**C**,**D**) development. Plant leaves were brushed with the solutions for 14 days (**A**) or sprinkled with 5 mL solution to the root at early seedling (**B**,**C**) or with 10 mL solutions for 10 days at a later stage (**D**). CON = non-treated control. On picture (**D**), the red arrows show the small leaves close to the soil level induced by BA treatment.

To compare the effects of BA and kinetin on plant phenotype, we treated plants with the two cytokines by various ways at different developmental stages of *Arabidopsis* and tobacco. Brushing of *Arabidopsis* leaves with BA solution inhibited the development of the flowering stem and induced thickening and curling of the central rosette leaves. In contrast, kinetin treatment rather promoted flowering, and no rosette leaf thickening or curling was observed (Figure 1A). In addition, when BA solution was sprinkled at an early developmental stage to the roots, the development of both *Arabidopsis* and tobacco plants was strongly inhibited, while a similar application of kinetin had no visible effect (Figure 1B,C). The application of BA solution to tobacco at later developmental stage by watering caused a rapid formation of small leaves close to the soil level (Figure 1D), while the same application of kinetin had no or weak effect (data not shown).

When older tobacco plants were treated with different doses of BA, it was observed that the effect of lower BA doses was weaker. Meanwhile, a 3 × 30 mL dose of saturated BA solution strongly (Figure 1D), 2 × 30 mL doses slightly, and the 1 × 30 mL dose did not induce the rapid formation of small leaves close to the soil level. The effect of kinetin compared to BA was weaker because kinetin solution at 3 × 30 mL dose caused a slight formation of small leaves at the soil level, while 2 × 30 mL and 1 × 30 mL doses caused no formation of small leaves at the soil level at all (data not shown).

**Figure 2:**
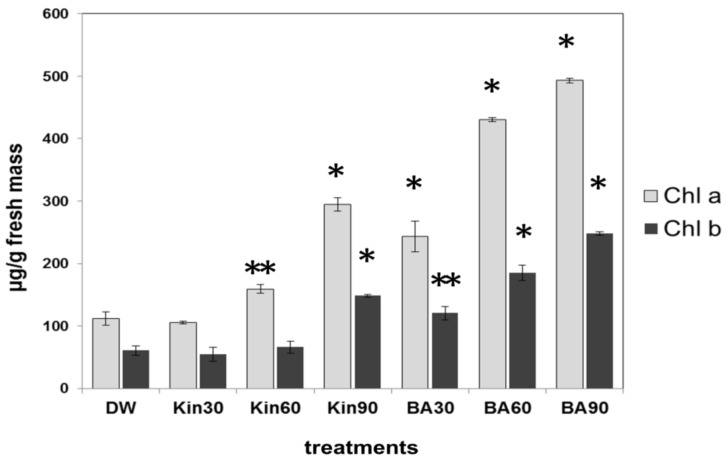
Chlorophyll a and b contents (μg/g fresh mass) of leaves from Xanthi tobacco plants pre-treated with distilled water (DW), 30 mL (1 × 30), 60 mL (2 × 30), or 90 mL (3 × 30) of saturated benzyladenine (BA) or kinetin (KIN) solutions. Chlorophyll contents were determined five weeks after the last treatment with the cytokinin solutions. Bars represent mean ± standard deviations of three replicates of a representative experiment from two independent experiments with similar results. The significant difference from the corresponding water-treated control was determined (* *p* < 0.005, ** *p* < 0.05, two-tailed *t*-test).

Since we found that high doses of cytokinins were able to inhibit the senescence of older tobacco leaves, the chlorophyll content as a senescence marker of these leaves was determined. The chlorophyll content of the second fully developed leaves (from the ground) of tobacco plants showed correlation with the amount of the applied cytokinins (Figure 2). Compared with kinetin, BA was more efficient in the retardation of both chlorophyll a and b degradation, but the highest dose of kinetin (3 × 30 mL) was more effective than the lowest doses of BA (1 × 30 mL). The lowest dose of kinetin (1 × 30 mL) had no inhibitory effect on chlorophyll degradation compared to control, water-treated tobacco leaves. 

### 3.2. Benzyladenine and Kinetin Have Different Effect on Biotic Stress Tolerance

**Figure 3:**
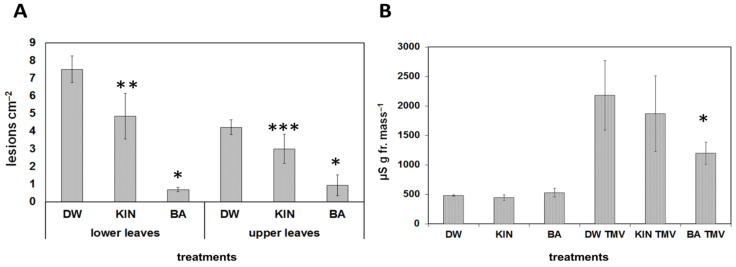
Effect of water (DW), kinetin (KIN), or benzyladenine (BA) pre-treatment on TMV infection on Xanthi nc. tobacco leaves. Infection was carried out 2 days after the last treatment. (**A**) Number of lesions on lower and upper TMV-infected leaves. * *p* < 0.001, ** *p* < 0.05, *** *p* < 0.1 two-tailed *t*-test. (**B**) Ion leakage from TMV-infected leaf discs of water (DW), kinetin (KIN), or benzyladenine (BA) pre-treated tobaccos. Bars represent the mean ± standard deviations of three replicates of a representative experiment from three independent experiments with similar results. The significant difference from the corresponding water-treated control was determined (* *p* < 0.05, two-tailed *t*-test).

Pre-treatments with the BA or kinetin had a different effect on the reactions of plants to pathogen infections as well. *Tobacco mosaic virus* (TMV) induced necrotic lesions in the incompatible reactions on leaves of *Nicotiana tabacum* cv. Xanthi nc tobacco. The lesion number caused by TMV infection was strongly and significantly reduced both on lower or upper leaves as compared to water-treated leaves if tobacco plants were sprinkled every day for 10 days with BA solution before infection. On the other hand, pre-treatment with kinetin solution had a more slight effect on lesion number both on lower and upper leaves as compared to the control water-treated plants (Figure 3A).

**Figure 4:**
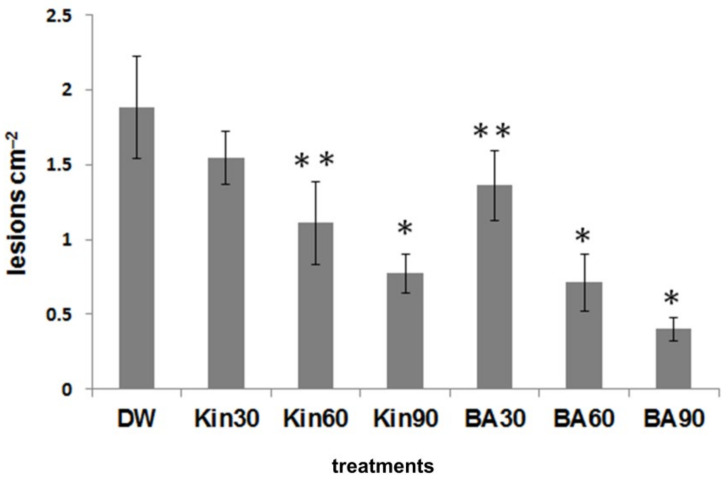
Effect of pre-treatments with various doses (30 mL (1 × 30), 60 mL (2 × 30), and 90 mL (3 × 30)) of saturated benzyladenine (BA), kinetin (KIN), or distilled water (DW) on the number of TMV lesions on Xanthi tobacco leaves (3 dpi). Infection was carried out 2 days after the last treatment. Bars represent mean ± standard deviations of three replicates of a representative experiment from two independent experiments with similar results. The significant difference from the corresponding water-treated control was determined (* *p* < 0.001, ** *p* < 0.05, two-tailed *t*-test).

Ion leakage correlates well with the membrane damage during necrosis development, and it is a good marker of cell damage; therefore, conductivity measurements were carried out as well. Similarly to lesion numbers, ion leakage from TMV-infected tobacco leaf discs was significantly reduced when the plants were pre-treated with BA as compared to water-treated controls, but no significant difference was found when tobacco plants were pre-treated with kinetin solution (Figure 3B). The effect of different doses of the two cytokinins on TMV-induced lesions on Xanthi tobacco leaves was also tested. We found that the higher the amount of BA or kinetin, the less the number of TMV-induced lesions. Again, BA had a stronger effect than kinetin, but the highest dose of kinetin was more effective at suppressing TMV lesions than the lowest dose of BA (Figure 4).

**Figure 5:**
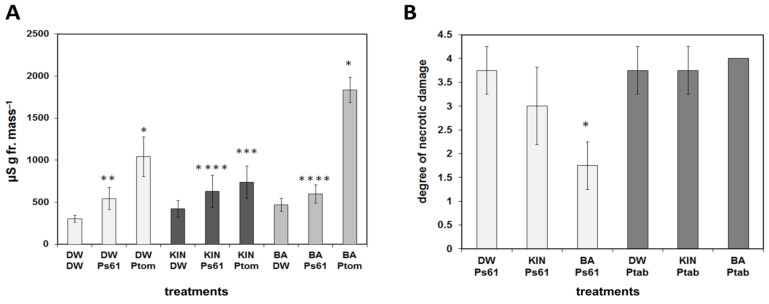
(**A**) Electrolyte leakage from water (DW), kinetin (KIN), or benzyladenine (BA) pre-treated *Arabidopsis* leaves at 3 dpi after inoculation with water (DW), incompatible *P. s. pv. s.* 61 (Ps61), or compatible *P. s. pv. t.* DC3000 (Ptom) bacteria. * *p* < 0.01, ** *p* < 0.05, *** *p* < 0.1, **** *p* < 0.2 two-tailed *t*-test. (**B**) The reaction of water (DW), kinetin (KIN), or benzyladenine (BA) pre-treated tobacco plant leaves to incompatible *P.s. pv. s.* 61 (Ps61) or compatible *P.s. pv. tabaci* (Ptab) bacteria at 3 dpi as evaluated by scoring (from 0 to 4) the necrotic damage caused by bacterial suspensions injected into tobacco leaves. W = water. * *p* = 0.001 two-tailed *t*-test. Bars represent mean ± standard deviations of three replicates of a representative experiment from three independent experiments with similar results. The significant difference from the corresponding water-treated control was determined.

Furthermore, the effect of pre-treatments with BA or kinetin on the reaction of plants to incompatible and compatible bacteria gave similar but somewhat surprising results. Electrolyte leakage from water, BA, or kinetin pre-treated *Arabidopsis* leaves after inoculation (brushing with bacterial suspension) with incompatible *P.s.* pv. *s.* 61 or compatible *P.s*. pv. *t.* DC3000 bacteria were measured 3 days after infection (Figure 5A). It is noteworthy that BA and kinetin treatments alone increased ion leakage. In water pre-treated leaves, the incompatible bacteria caused a significant 78% increase in conductivity (leakage of electrolytes) compared to its non-infected control, indicating the damage of plant cell membranes during HR in control leaves. In kinetin pre-treated leaves, the conductivity increase caused by the incompatible bacteria was smaller (49% increase as compared to its kinetin-treated non-infected control), and it was almost abolished in BA pre-treated plants (only 27% increase as compared to its non-infected control) (Figure 5A). Thus, similarly to TMV infection, the cell death and necrosis suppressor effect of BA was strong, while that of kinetin was much less expressed. On the other hand, as compared to the incompatible one, the compatible hemi-biotrophic bacteria caused much stronger leakage of electrolytes 3 dpi, which is probably due to vigorous multiplication and leaf spot disease-inducing ability of the bacteria in susceptible leaves (Figure 5A). However, surprisingly, while in BA, pre-treated leaves infection with *P.s*. pv. *t.* DC3000 bacteria increased ion leakage by 293% (compared to BA pre-treated non-infected leaves) and in water-treated control leaves by 241% (compared to non-infected control), in kinetin pre-treated leaves infection with *P.s*. pv. *t.* DC3000 bacteria increased ion leakage only by 75%. Thus, in this respect, BA and kinetin treatments had the opposite effect on the reaction of *Arabidopsis* leaves to compatible bacteria since BA increased and kinetin decreased ion leakage.

In tobacco, by measuring the reaction of leaves to incompatible *P.s.* pv. *s.* 61 or compatible *P.s.* pv. *tabaci* bacteria, we got similar results, although the injection of compatible bacteria into tobacco leaves caused much stronger damage (disease development) than brushing *Arabidopsis* leaves with the same bacterial suspension. Therefore, the compatible bacteria caused total necrotization (score 4) of the injected leaf area in BA treated and only slightly less strong necrosis in water or kinetin-treated plants 3 days post inoculation (Figure 5B). On the other hand, the hypersensitive necrotic response caused by the incompatible bacteria was significantly suppressed again in leaves of BA pre-treated (46.7%) and not significantly in the kinetin pre-treated (80%) tobacco plants as compared to the water-treated (100%) ones (Figure 5B).

**Figure 6:**
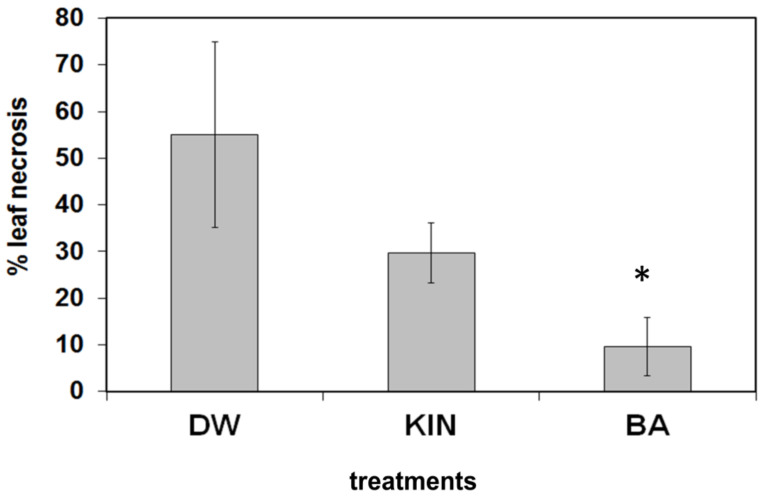
Effect of water (DW), kinetin (KIN), or benzyladenine (BA) pre-treatment on *Botrytis cinerea* infection on tobacco leaves. Pre-treated leaves were removed from tobacco plants and infected with a 0.5 cm in diameter agar culture disk of the pathogen in glass Petri dishes After 5 days, the extent of pathogen-induced leaf necrosis relative to the total leaf area was determined. Bars represent mean ± standard deviations of three replicates of a representative experiment from three independent experiments with similar results. The significant difference from the corresponding water-treated control was determined (* *p* = 0.019 two-tailed *t*-test).

In order to learn the effect of the above cytokinin pre-treatments on the reaction of plants to necrotrophic fungi, leaves from BA or kinetin-treated tobacco plants were infected with agar discs from *Botrytis cinerea* 5-day-old culture and kept on wet filter paper in a Petri dish. As it can be seen from Figure 6, the pathogen caused very severe necrotic symptoms on leaves from water-treated plants, somewhat less on leaves from kinetin-treated plants, and significantly less on leaves from BA-treated plants, as is quantified by percentage of leaf necrosis. 

### 3.3. Benzyladenine Causes Stronger Transcriptomic Alterations Than Kinetin in Arabidopsis Leaves

In order to obtain a wider picture of the plant responses to different cytokinin treatments and to find some answer for the possible background of different responses, we performed transcriptomic experiments. For gene expression measurements, *Arabidopsis* plants were treated with leaf brushing with BA or kinetin, and water-treated plants were used as a control. The transcriptomic changes were measured by Agilent Arabidopsis (V4) Gene Expression Microarray (4 × 44K). Gene expressions data of the microarray assays confirmed the diverse results of development and pathogen stress in BA and kinetin-treated plants.

**Figure 7:**
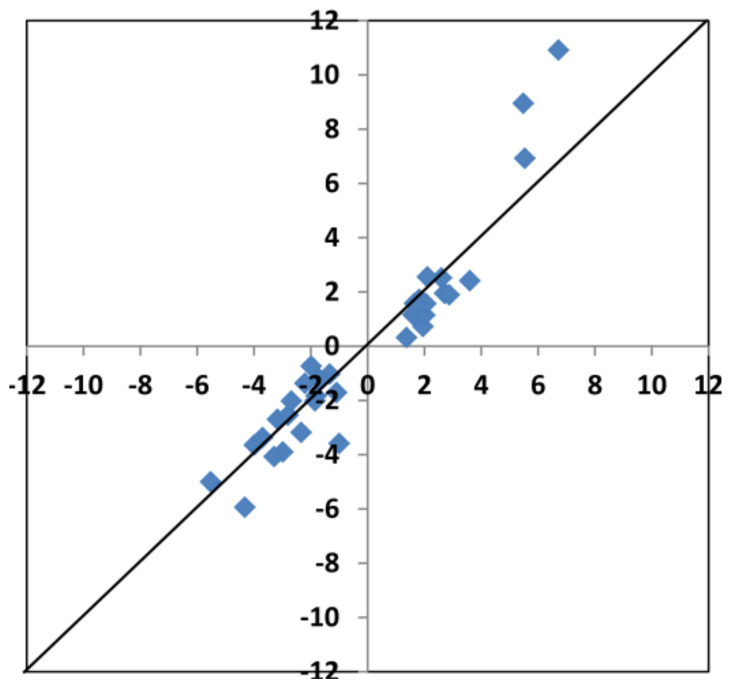
Comparison of expression changes of selected *Arabidopsis* genes detected with microarray and real-time RT-PCR after kinetin (KIN) or benzyladenine (BA) treatments. X-axes show the average log_2_ transcription activation or repression detected with microarray (up or downregulated in kinetin or benzyl adenine treatments leaves compared to water-treated control). Y-axes show the average log_2_ transcription activation or repression detected with real-time RT-PCR (up or downregulated in kinetin or benzyladenine treatments leaves compared to water-treated control).

**Figure 8:**
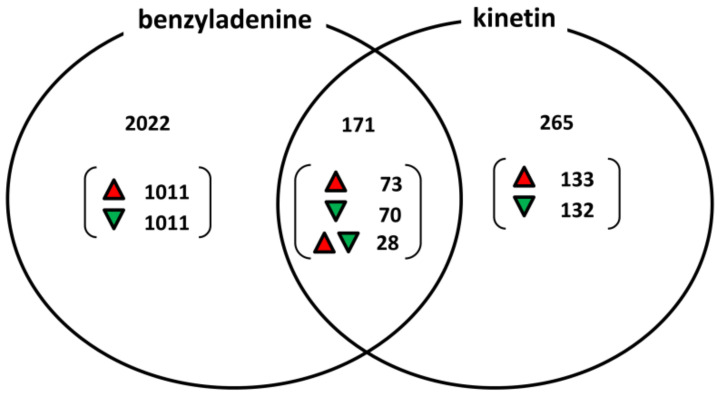
Number of up and downregulated genes after benzyladenine or kinetin treatments in *Arabidopsis* leaves. The figure shows the results of the microarray experiments. Genes activated or repressed significantly at least two times compared to water-treated control was indicated. 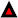 = upregulated genes, 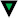 = downregulated genes, 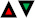 = genes regulated by the treatments to opposite directions.

After data processing, the results showed that at a significance level of adj.P.Val < 0.05, only the BA treatment caused transcriptomic alterations compared to water-treated control. However, at a slightly lower significance level (adj.P.Val < 0.07), the kinetin treatment also showed many gene expression alterations. To confirm the reliability of the expression data at adj.P.Val < 0.07, we performed qPCR measurements with 24 representative genes. We choose three representative genes from the eight group of genes (BA repressed/kinetin activated, BA activated/kinetin repressed, both BA and kinetin activated, both BA and kinetin repressed, only BA activated, only BA repressed, only kinetin activated, only kinetin repressed). The results of the qPCR tests practically corresponded to the microarray data. As it can be seen from  Supplementary Table S2 and Figure 7, the qPCR results showed the same tendency and similar values as the microarray experiments (Pearson correlation coefficient value of R is: 0.9523). Since the gene expression data were reliable at adj.P.Val < 0.07, the results were further analysed at this significance level. BA treatments upregulated 1011 genes and downregulated 1011 genes, which were not changed after kinetin treatment (Figure 8).

In addition, not only the number of affected genes but the magnitude of the changes was much larger after BA than kinetin treatments (Supplementary Table S1). While the highest activation changes were about 100× induction among the BA-specific genes and the average induction was about 3.9×, the highest activation after kinetin treatment was only 7.3× and the average induction was about 2.4×. However, in spite of the much stronger effect of BA, we found 133 up and 132 downregulated genes whose activity was specifically modified by kinetin treatment and was not changed by BA (Figure 8). It is noteworthy that BA and kinetin treatment changed gene expressions into the same direction only in relatively few cases (73 upregulated and 70 downregulated genes); however, most interestingly, there were 28 genes where BA and kinetin treatments caused changes of gene expressions into the opposite directions (Figure 8, Table 1). The whole list of differentially regulated genes can be found in Supplementary Table S1.

Among the genes specifically activated by BA, the most upregulated (about 100 times) was a bifunctional inhibitor/lipid-transfer protein/seed storage 2S albumin superfamily protein gene, while the most downregulated (more than 45 times) was a jasmonate-zim-domain protein gene. Furthermore, in addition to the other three bifunctional inhibitor/lipid-transfer protein/seed storage 2S albumin superfamilies, the most strongly upregulated genes by BA were a pathogenesis-related protein 1 (PR1) and defensin-like protein, LRR-receptor-like protein kinase, beta 1,3-glucanase, and a putative chitinase genes. It is also noteworthy that a cytokinin dehydrogenase 3 and three two-component response regulator ARR genes (negative regulators of cytokinin signals) were strongly upregulated by BA (Supplementary Table S1). Among the most downregulated genes by BA were three cytochrome P450 (family 94), lipoxygenase 3 and 4, two cold-regulated proteins, a pathogenesis-related thaumatin family protein gene, and two bifunctional inhibitor/lipid-transfer protein/seed storage 2S albumin superfamily protein genes.

The most upregulated genes by both BA and kinetin were a gibberellin 20 oxidase 1 mRNA, three SAUR-like auxin-responsive protein mRNA, a gibberellin-regulated protein mRNA, three expansin (A8, A10, and an A11), a putative pectate lyase, as well as a dehydration-responsive element-binding protein 1A genes. The most downregulated genes by both BA and kinetin were two ethylene-responsive transcription factors, a heat stress transcription factor, an abscisic acid receptor, three chaperone DNaJ-domain containing proteins, a calcium-binding protein, and calmodulin-like protein genes.

The most induced genes by kinetin and not BA treatments were Li-tolerant lipase 1, two GDSL esterase/lipase, CBL-interacting protein kinase 5, a glucose-methanol-choline (GMC) oxidoreductase-like protein, a PATATIN-like 9 (phospholipase), two putative cinnamyl alcohol dehydrogenase 9, and dehydration-responsive element-binding protein 3 genes. The most suppressed genes by only kinetin treatments were wall-associated receptor kinase-like 10, proline dehydrogenase 2, heat shock protein 70, as well as class V 15.4, defence-like protein, and abscisic acid receptor PYL6 genes (Supplementary Table S1).

To find common and specific gene responses and plant processes induced by kinetin or BA treatments, the gene expression data were also compared by GO enrichment [36] and by MapMan analysis [37]. The difference of kinetin and BA-specific responses were supported by these comparisons and besides showed some common features of these treatments as well. The results pointed out that both treatments induced remarkable transcriptional reprogramming of the plant cells, but the BA had a stronger effect on transcription. The genes connected to the regulation of transcription were one of the most significantly enriched terms among BA-activated genes and among the BA-repressed genes, this term was also significantly enriched (Table 2, Table 3).

The kinetin treatments also induced significant enrichment among upregulated genes related to gene regulation but not among downregulated ones (Table 4, Table 5). According to enrichment analysis, high numbers of up or downregulated transcription factors were showed by MapMan results (data not shown). Other typical common features of kinetin and BA on gene expression were that both treatments influenced the different hormone-related responses. Interestingly, only BA-activated genes had a significant impact on cytokinin-induced processes (Table 2), but both treatments had a significant effect on auxin and gibberellin-related stimulus (Table 2, Table 4). In addition, BA influenced some well-known defence-related hormone response such as salicylic acid, jasmonic acid, and ethylene responses in both activated and repressed genes (Table 2, Table 3). It is remarkable that BA repressed several consecutive steps of JA synthesis, but in the case of kinetin treatments, this was not discernible (Supplementary Figure S1).

The enrichment analysis also revealed that both treatments activate cell wall loosening-related genes that may connect to cell growth, whose genes are also upregulated by the two treatments (Table 2, Table 4). Based on gene expression results, there were some other general processes that influenced by BA but not kinetin, such as protein synthesis and photosynthesis. BA significantly affected ribosome biogenesis and activated many ribosome structural genes, while repressed several genes involve in photosynthesis light reaction (Table 2, Table 3). Other noteworthy differences can be seen in relation to the phenylpropanoid/flavonoid/anthocyanin biosynthetic processes. Although both treatments significantly enriched upregulated genes related to the phenylpropanoid biosynthetic process (Table 2, Table 4), in the case of kinetin, the direction of the changes was more uniform, since all detected genes were activated (Supplementary Figure S2). In contrast, the picture after BA treatment is more complex, because mainly, the early steps of phenylpropanoid synthesis were upregulated and almost all flavonoid/anthocyanin synthesis genes were repressed (Supplementary Figures S2 and S3). Moreover, some genes related to anthocyanin biosynthesis are part of the common genes regulated in the opposite direction by BA and kinetin (repressed by BA and activated by kinetin (Table 1). Finally, the results also showed that the plant defence-related processes are more affected by BA than kinetin. After the BA treatment, the defence-related genes were enriched in both the activated and repressed genes such as genes related to resistance against bacteria and fungus, which may refer to complex changes induced by BA in plant cells (Table 2, Table 3). On the other hand, kinetin did not cause significant enrichment defence-related changes among upregulated genes at all (Table 4).

## 4. Discussion

Although there are a large amount of data about the effect of cytokinins on various aspects of plant development [38,39,40,41], the number of publications comparing the effect of two different cytokinins on plants are limited. Our data provide new and somewhat astonishing data on the different effect of two structurally similar cytokinins. We obtained the first surprising results when *Arabidopsis* leaves were brushed with the solutions of BA, kinetin, or water. BA solution inhibited the development of flowering stem, while kinetin treatment rather promoted it as compared to the water-treated ones (Figure 1A). As regards the mechanisms of the effect of cytokinins on plant growth, it is noteworthy that cytokinins are considered as inhibitors of root growth and promoters of shoot growth [1]. Although *Cajanus cajan* L. plants showed a significant increase in branch number, leaf number, leaf area, and seed mass to kinetin treatment [42], our results suggest that *Arabidopsis* is much more sensitive to BA, and its effect is “too strong”, therefore inhibitory, while the sensitivity to kinetin is much less, and this treatment seemed to stimulate development of the flowering stem. The different effects of these two cytokinins on plants were further supported by experiments in which test solutions were sprinkled to the root at the early stages of the seedling. BA strongly inhibited the development of both *Arabidopsis* and tobacco plants, whereas kinetin had no such effect. (Figure 1B,C). Furthermore, the general observation of the experiments was that diluted BA had little or no effect on plant phenotypes compared to the saturated solution, and kinetin did not cause or could cause a slight phenotypic change at most only as a saturated solution. These changes had been seen in the cases of *Arabidopsis* flower development and tobacco small leaves formation induction as well (data not shown). Accordingly, Argueso et al. [3] showed that high concentrations of cytokinin lead to increased defence responses to a virulent oomycete pathogen through a process that is dependent on salicylic acid (SA) accumulation and activation of defence gene expression. However, treatment with lower concentrations of cytokinin resulted in increased susceptibility. Similar results are seen in response to wheat to powdery mildew [43]. However, we have to point out that in our experiments, the lower molar concentration of BA had a much stronger effect than a higher molar concentration of another cytokinin kinetin. The chlorophyll content of the second oldest leaves of tobacco plants also showed correlation with the amount of the applied cytokinins. BA was more efficient in the retardation of both chlorophyll a and b degradation, but higher doses of kinetin were more effective than the lowest doses of BA (Figure 2), and the lowest doses of kinetin had no inhibitory effect on chlorophyll degradation as compared to the control water-treated tobaccos leaves. All these data indicate that the senescence inhibiting and developmental effect of both cytokinins depends on their doses of application. In addition, it is known that different cytokinins have different binding affinities for the histidine kinases, which function as their cognate receptors in plants. Such differences have been well documented biochemically for *Arabidopsis*. For example, BA has a 10-fold lower affinity for HK receptors in *Arabidopsis* than the naturally occurring trans-zeatin [44]. Thus, the intensity of signal transduction resulting from the use of similar concentrations of different cytokinin species can be different in plant cells (due to their different affinity for cognate receptors). This is in fact corroborated by the gene expression studies performed in the present study: While the foliar applications of BA in the mM range had a clear effect on upregulating cytokinin-responsive genes, an even 30% higher concentration of kinetin did not do so.

It is noteworthy that in our experiments, in addition to plant development, BA had a much stronger effect on drought tolerance than kinetin treatment, especially in the case of *Arabidopsis* plants (data not shown). In accordance, there are reports about the protecting effect of cytokinin treatments to drought, salt, and generally abiotic stresses [42,45,46,47], which is probably due to the elevated stress tolerance of juvenile plant tissues [14]. In addition, we should emphasize that the hormonal control of plant development and stress adaptation is the outcome of a complex network of multiple synergistic and antagonistic interactions between various plant hormones [48,49,50,51,52]. 

In spite of the ever-increasing number of publications on the mechanisms of plant resistance to pathogens, there are still many open questions [15,16,17]. The importance of plant hormones and especially cytokinins in plant disease development and in plant immunity is discussed in several papers [18,53,54,55,56,57]. The stronger effect of BA treatment was also experienced in the case of pathogen-induced stresses; however, in these cases, the picture was complicated by the effect of these treatments on the *in planta* multiplication of the pathogens. Namely, the hypersensitive necrotic symptoms are not always in correlation with resistance to the pathogen [58]. BA treatment of tobacco plants strongly reduced TMV-induced lesion development on leaves, while kinetin treatment weakly reduced TMV-induced lesion development on leaves, which was also shown by the conductivity assay (Figure 3). It is also noteworthy that in the case of all treatments, the number of lesions was significantly reduced in the younger upper than older lower tobacco leaves. However, in our previous publication, we proved that the number of lesions caused by TMV infection and virus multiplications are not always in correlation [56]. Accordingly, the HR caused by the incompatible *P. s*. pv. *s.* 61 bacteria was significantly reduced by BA and much less by kinetin pre-treatment in tobacco leaves. However, the compatible *P. s.* pv. *tabaci* caused leaf necrosis to a similar extent on water, kinetin, or BA pre-treated plants (Figure 5B). These results are in agreement with our previous data where cytokinin-overproducing tobacco showed elevated tolerance to incompatible *P. syringae*-induced HR but not to the necrosis caused by compatible *P. s.* pv. *tabaci*. Moreover *P. s.* pv. *tabaci* showed higher multiplication in cytokinin-overproducing than in the control not transformed tobacco leaves [23]. In the case of *Arabidopsis*, we obtained similar results. The incompatible bacteria caused the largest membrane damage (ion leakage) in water-treated, while it caused the smallest in BA-treated plants compared to their non-infected controls. Connection of cytokinin and the cell death process was further supported by the fact that the protein Bax Inhibitor-1 (BI-1), a negative regulator of plant programmed cell death (PCD), was upregulated following treatments with cytokinins [59].

On the other hand, the compatible bacteria induced the strongest leakage of ions from leaves of the BA-treated plants (Figure 5A). This phenomenon is most probably due to the stronger multiplication of compatible bacteria in juvenile tissues, as we found earlier [23,30]. However, it is noteworthy that BA and kinetin treatments had the opposite effect on the reaction of *Arabidopsis* leaves to compatible bacteria, since BA increased membrane damage (ion leakage) but kinetin decreased membrane damage (ion leakage) compared to the water-treated ones (Figure 5A).

The picture of the reactions of the treated plants to the necrotrophic pathogen *Botrytis cinerea* infection was clearer. BA treatment suppressed the necrotic disease symptoms and membrane damage (Figure 6). Since cytokinins inhibit leaf senescence [60,61] and necrotrophic pathogens prefer the senescent older plant tissues, therefore, not only the necrotic symptoms but the development of the pathogen is also suppressed in juvenile tissues [23,28].

Regarding the mechanisms, we think that the enhanced tolerance of juvenile plant tissues to necrotic pathogenic stress is due to, partly at least, to their more stable membranes [62,63] and elevated antioxidant capacity [14,22,23,24], as we proved also in the case of transgenic ferritin overproducing tobacco by inhibiting the formation of the most harmful reactive oxygen, the hydroxyl radical [64].

Our gene expression data confirmed the diverse effect of kinetin and BA on *Arabidopsis* plants. There are several papers dealing with gene expressions of cytokinin-treated *Arabidopsis* [5,6,65,66,67]. However, as it is explained by Bhargava et al. [68], it is difficult to compare the results of various papers because of different lab/growth and treatment-specific effects. To check how our transcriptomic results relate to previous cytokinin-induced experiments, we compared these results with some gene lists obtained from publications that summarise several microarray experiments (Supplementary Table S3). It was clear from the comparisons that the results also depended on the gene expression platforms used for transcriptomics. Brenner et al. [65,67] published the TOP 25 cytokinin-activated genes of meta-analysis of Affymetrix ATH1 and CATMA microarrays. From the TOP 25 ATH1 genes, 17 (68%) were found in our BA-treated samples, and from the TOP 25 CATMA array, 12 genes (48%) were found in our BA-treated samples. The kinetin treatments have only limited overlapping with these TOP 25 gene lists, since one and two genes were common with ATH1 and CATMA genes, respectively. Bhargava et al. [68] published two more detailed lists of cytokinin-regulated genes. One of these lists is based on a meta-analysis of 13 ATH1 type array experiments and produced a so-called golden list of cytokinin-regulated genes of *Arabidopsis* that contains 226 genes (158 upregulated and 68 downregulated). From these 226 genes, about 35% (78) were influenced significantly in our BA-treated and only 4% (9) were influenced significantly by kinetin. The other cytokinin-regulated list obtained from analysis of an RNA-seq based method contains 375 significantly regulated genes (226 upregulated and 149 downregulated) and about 28% (104) were influenced significantly in our BA-treated and again only 4% (16) were influenced significantly by kinetin. The low level of overlapping between our kinetin treatment and the results of cytokinin-modified genes list obtained by meta-analysis could be due to using BA or zeatin in the experiments for meta-analyses.

It is difficult to pick up individual genes from the almost 2500 transcriptionally altered genes that are definitely responsible for the changes in response to cytokinin treatments. However, some tendencies in the gene expression changes can be recognized. First of all, BA solution had stronger effect than kinetin solution not only on the biotic stress tolerance of *Arabidopsis* plants but also on gene expressions. The larger amount of up and downregulated genes by BA treatments can be probably explained by the higher binding affinities for receptors. However, in spite of the more than 2000 genes that exclusively changed their expression after BA treatment, there were 265 up or downregulated genes that changed their expressions only by kinetin treatment. More surprisingly, there were only 73 up and 70 downregulated genes that altered their expression in the same direction after both cytokinin treatments. These latter results, together with the 28 genes that change their expressions into the opposite direction after BA and kinetin treatments, emphasise the diverse effect of these cytokinins on plant processes (Figure 8).

One group of genes that we should recognize are hormone-related genes. Both BA and kinetin treatments upregulated a gibberellin oxidase gene (AT4G25420), a gibberellin-regulated gene (AT2G14900), and three SMALL AUXIN UPREGULATED (SAUR)-like auxin-responsive genes (AT4G34770, AT4G34790, AT3G03820), while they downregulated two ethylene-responsive transcription factors (AT2G20880, AT1G22810) and an abscisic acid receptor gene (AT5G05440). Furthermore, a cytokinin dehydrogenase 3 (AT5G56970) and three two-component response regulator ARR genes (negative regulators of cytokinin signals, AT5G62920, AT1G74890, AT3G48100) were strongly upregulated, while a jasmonate-zim-domain gene (AT2G34600) was strongly downregulated by BA. Additionally, an abscisic acid receptor mRNA gene (AT2G40330) was downregulated by kinetin treatment. All these data strengthened the hypothesis on the concerted action of the network of plant hormones [5].

The other interesting group consists of the pathogenesis-related genes. BA treatment strongly upregulated a pathogenesis-related protein 1 (PR1, AT2G14610), a defensin-like protein (AT1G19610), an LRR-receptor-like protein kinase (AT1G51890), a beta 1,3-glucanase (PR2, AT3G57260), and a putative chitinase gene (AT2G43570). Interestingly, a pathogenesis-related thaumatin family gene (AT4G36010) was downregulated by BA treatment. On the other hand, kinetin treatment did not upregulate pathogenesis-related proteins but downregulated a defence-like protein mRNA.

An additional group is formed by stress-related genes. BA suppressed two cold-regulated protein genes (AT2G42540, AT5G42900), while genes At5MAT (AT3G29590) and TT8 (AT4G09820) involved in flavonoid/anthocyanin metabolism were downregulated by BA and upregulated by kinetin. It is noteworthy that a dehydration-responsive element-binding gene (AT4G25480) was among the most upregulated genes by both BA and kinetin, which can be related to an increase in draught tolerance. Among the most downregulated genes by both BA and kinetin was a heat stress transcription factor gene (AT2G26150). Kinetin treatment alone upregulated a dehydration-responsive element-binding gene (AT2G26150) but downregulated a heat shock protein 70 gene (AT5G02490). Interplay between biotic, abiotic, and hormone-related responses were also experienced previously by analysing transcriptomic data in different plant pathogen interactions [69,70]. It is also noteworthy that kinetin treatment affected lipase and phospholipase genes. Accordingly, the most induced genes by kinetin and not BA treatments were Li-tolerant lipase (AT3G04290), two GDSL esterase/lipase (AT5G33370, AT4G28780), and a PATATIN-like 9 (phospholipase, AT3G63200).

The other group of genes induced by kinetin and not BA treatments consisted of oxido-reductase enzymes, such as a glucose-methanol-choline (GMC) oxidoreductase-like gene (AT1G12570) and putative cinnamyl alcohol dehydrogenase 9 genes (AT4G39330). In addition, among the most suppressed genes by only kinetin treatment was a proline dehydrogenase 2 gene (AT5G38710). BA repressed allene oxide cyclase 1 (AT3G25760).

Naturally, we could separate the other group of genes that show characteristic changes after BA or kinetin treatment, but we think that the above selected groups of genes are possibly involved in the various responses of the two cytokinin-treated plants.

In conclusion, we obtained different, sometimes opposite, effects of kinetin and benzyladenine treatments on development and tolerance to virus, bacteria as well as to fungal infection of *Arabidopsis* and tobacco plants. Generally, BA treatment had a much stronger effect on the development and stress tolerance of both plants than treatments with kinetin, which were reflected in the changes in gene expression profiles of *Arabidopsis* as reactions to treatments with the two cytokinins. The stronger protective effect of BA to necrotic stresses is probably due to its stronger effect on plant tissues to inhibit senescence, as was shown by the chlorophyll contents in leaves of the cytokinin-treated plants. The practical application of the BA-induced biotic and abiotic stress tolerance needs further investigations, which is under progress.

## Figures and Tables

**Table 1:** *Arabidopsis* genes regulated by kinetin (Kin) or benzyladenine (BA) in opposite directions.

Array ID ^a^	Arabidopsis TAIR Code ^b^	Fold-Change ^c^ Kin BA		Similarity, Function
A_84_P13657	AT3G29590	2.0	−1.8	malonyl-CoA:anthocyanidin 5-O-glucoside-6’-O-malonyltransferase ^d^
A_84_P18522	AT4G09820	2.0	−1.0	transcription factor TT8
A_84_P808177	AT5G59320	1.7	−4.3	non-specific lipid-transfer protein 3
A_84_P12100	AT5G25980	1.5	−1.0	myrosinase 2 (thioglucoside glucohydrolase)
A_84_P842527	AT5G42800	1.5	−2.5	DFR (dihydrokaempferol 4-reductase)
A_84_P20780	AT1G03495	1.4	−1.8	HXXXD-type acyl-transferase family protein
A_84_P13548	AT1G07430	1.3	−1.2	protein phosphatase 2C 3
A_84_P253555	AT4G14090	1.2	−2.2	anthocyanin 5-O-glucosyltransferase
A_84_P813812	AT3G08770	1.2	−1.8	lipid-transfer protein 6
A_84_P12885	AT4G22880	1.1	−1.7	leucoanthocyanidin dioxygenase
A_84_P12954	AT4G39210	1.1	−1.0	glucose-1-phosphate adenylyltransferase large subunit 3
A_84_P804836	AT1G68530	1.0	−1.3	3-ketoacyl-CoA synthase 6
A_84_P750651	AT1G59930	−1.0	1.3	maternally expressed imprinted protein
A_84_P22866	AT1G20070	−1.0	1.5	uncharacterized protein
A_84_P860885	AT2G05540	−1.1	1.1	glycine-rich protein
A_84_P14446	AT2G19190	−1.1	3.6	FLG22-induced receptor-like kinase 1
A_84_P18423	AT3G47480	−1.2	1.8	putative calcium-binding protein CML47
A_84_P814710	AT1G14870	−1.3	1.9	cadmium resistance protein 2
A_84_P51580	AT1G72060	−1.4	1.0	serine-type endopeptidase inhibitor mRNA
A_84_P544532	AT1G58225	−1.5	1.4	uncharacterized protein
A_84_P14903	AT5G11920	−1.6	1.3	beta-fructofuranosidase
A_84_P10728	AT2G29110	−1.7	1.4	glutamate receptor 2.8
A_84_P511702	AT1G53625	−1.7	1.5	uncharacterized protein
A_84_P17225	AT2G47190	−2.0	1.2	R2R3 MYB DNA binding domain transcription factor
A_84_P827683	AT1G65510	−2.2	1.0	uncharacterized protein
A_84_P586318	AT1G65845	−2.2	1.2	uncharacterized protein
A_84_P737368	AT1G36622	−2.3	2.1	uncharacterized protein

^a^ Agilent Arabidopsis (V4) Gene Expression Microarray, 4 × 44K identifiers. ^b^ Arabidopsis TAIR gene identifiers. ^c^ gene expression in log_2_ transformed form compared to water-infiltrated control (average of three repeats), red and green backgrounds show up- and down-regulated genes, respectively. ^d^ genes involve in anthocyanin synthesis were highlighted by grey background.

**Table 2:** Enrichment results of genes activated by benzyladenine.

Biological Processes (998 Genes)			
GO_acc	Term	Number of Genes ^a^	FDR ^b^
GO:0045449	regulation of transcription ^c,d^	93	2.70 × 10^−7^
GO:0009735	response to cytokinin stimulus	16	1.70 × 10^−6^
GO:0009739	response to gibberellin stimulus	20	2.70 × 10^−6^
GO:0006260	DNA replication	17	4.00 × 10^−6^
GO:0010876	lipid localization	9	5.90 × 10^−6^
GO:0009733	response to auxin stimulus	30	7.20 × 10^−6^
GO:0006949	syncytium formation	7	4.40 × 10^−5^
GO:0009751	response to salicylic acid stimulus	20	5.90 × 10^−5^
GO:0009828	plant-type cell wall loosening	8	0.0011
GO:0042742	defence response to bacterium	16	0.0016
GO:0009723	response to ethylene stimulus	17	0.0018
GO:0009699	phenylpropanoid biosynthetic process	14	0.0018
GO:0006979	response to oxidative stress	23	0.002
GO:0034976	response to endoplasmic reticulum stress	6	0.0026
GO:0009827	plant-type cell wall modification	8	0.0033
GO:0009791	post-embryonic development	37	0.0034
GO:0042254	ribosome biogenesis	18	0.004
GO:0006334	nucleosome assembly	8	0.0057
GO:0045454	cell redox homeostasis	9	0.0058
GO:0007623	circadian rhythm	9	0.007
GO:0009753	response to jasmonic acid stimulus	16	0.0076
GO:0010114	response to red light	9	0.0079
GO:0009933	meristem structural organization	7	0.016
GO:0009627	systemic acquired resistance	7	0.018
GO:0050832	defence response to fungus	10	0.018
GO:0009611	response to wounding	14	0.022
GO:0046686	response to cadmium ion	13	0.025
GO:0048509	regulation of meristem development	8	0.025
GO:0006874	cellular calcium ion homeostasis	5	0.036
GO:0051726	regulation of cell cycle	10	0.046
GO:0009825	multidimensional cell growth	6	0.047

^a^ number of genes associated with the GO term. ^b^ FDR < 0.05 was selected as significant enrichment. ^c^ terms common with kinetin-activated terms were highlighted by gray background. ^d^ terms common with BA-repressed terms were underlined.

**Table 3:** Enrichment results of genes repressed by benzyladenine.

	Biological Processes (930 Genes)		
GO_acc	Term	Number of Genes ^a^	FDR ^b^
GO:0009409	response to cold ^c^	47	7.20 × 10^−18^
GO:0009414	response to water deprivation	39	5.60 × 10^−17^
GO:0006970	response to osmotic stress	50	1.40 × 10^−16^
GO:0009611	response to wounding ^d^	35	1.00 × 10^−15^
GO:0010200	response to chitin	31	2.10 × 10^−15^
GO:0009737	response to abscisic acid stimulus	45	1.60 × 10^−14^
GO:0009753	response to jasmonic acid stimulus	31	8.20 × 10^−12^
GO:0051707	response to other organism	48	8.20 × 10^−10^
GO:0006952	defense response	53	1.10 × 10^−8^
GO:0009695	jasmonic acid biosynthetic process	9	1.40 × 10^−5^
GO:0006979	response to oxidative stress	27	1.40 × 10^−5^
GO:0019684	photosynthesis, light reaction	14	4.40 × 10^−5^
GO:0005983	starch catabolic process	6	0.00031
GO:0009723	response to ethylene stimulus	17	0.00068
GO:0045449	regulation of transcription	74	0.00069
GO:0006855	multidrug transport	10	0.00088
GO:0010876	lipid localization	6	0.0015
GO:0006863	purine transport	6	0.0021
GO:0010035	response to inorganic substance	19	0.0026
GO:0019761	glucosinolate biosynthetic process	7	0.0027
GO:0052482	cell wall thickening during defense response	5	0.0028
GO:0019762	glucosinolate catabolic process	5	0.004
GO:0052543	callose deposition in cell wall	5	0.004
GO:0042742	defense response to bacterium	14	0.004
GO:0042435	indole derivative biosynthetic process	7	0.0049
GO:0009718	anthocyanin biosynthetic process	5	0.0055
GO:0050832	defense response to fungus	10	0.0088
GO:0034641	cellular nitrogen compound metabolic process	26	0.0089
GO:0009626	plant-type hypersensitive response	7	0.01
GO:0005984	disaccharide metabolic process	6	0.02
GO:0000302	response to reactive oxygen species	8	0.026
GO:0006865	amino acid transport	7	0.026
GO:0009751	response to salicylic acid stimulus	13	0.028
GO:0009407	toxin catabolic process	6	0.038
GO:0009639	response to red or far red light	13	0.04
GO:0065008	regulation of biological quality	29	0.041
GO:0006796	phosphate metabolic process	45	0.045

^a^ number of genes associated with the GO term. ^b^ FDR < 0.05 was selected as significant enrichment. ^c^ terms common with kinetin-repressed terms were highlighted by gray background. ^d^ terms common with BA-activated terms were underlined.

**Table 4:** Enrichment results of genes activated by kinetin.

Biological Processes (199 Genes)			
GO_acc	Term	Number of Genes ^a^	FDR ^b^
GO:0009826	unidimensional cell growth	10	1.60 × 10^−5^
GO:0007169	transmembrane receptor protein tyrosine kinase signalling pathway	8	6.50 × 10^−5^
GO:0009828	plant-type cell wall loosening ^c^	5	0.00012
GO:0009825	multidimensional cell growth	5	0.00027
GO:0009699	phenylpropanoid biosynthetic process	7	0.00034
GO:0009813	flavonoid biosynthetic process	5	0.00092
GO:0048646	anatomical structure formation involved in morphogenesis	6	0.0012
GO:0006073	cellular glucan metabolic process	5	0.0022
GO:0006631	fatty acid metabolic process	7	0.004
GO:0009739	response to gibberellin stimulus	6	0.004
GO:0046148	pigment biosynthetic process	5	0.0055
GO:0006260	DNA replication	5	0.0063
GO:0009733	response to auxin stimulus	8	0.0094
GO:0045449	regulation of transcription	20	0.016
GO:0006468	protein amino acid phosphorylation	13	0.018
GO:0009611	response to wounding	5	0.036

^a^ number of genes associated with the GO term. ^b^ FDR < 0.05 were selected as significant enrichment. ^c^ terms common with BA-activated terms were highlighted by gray background.

**Table 5:** Enrichment results of genes repressed by kinetin.

Biological Processes (201 Genes)			
GO_acc	Term	Number of Genes ^a^	FDR ^b^
GO:0010200	response to chitin ^c^	10	3.50 × 10^−6^
GO:0006979	response to oxidative stress	13	6.60 × 10^−6^
GO:0009408	response to heat	6	0.013
GO:0009409	response to cold	8	0.019
GO:0010035	response to inorganic substance	7	0.031
GO:0051707	response to other organism	10	0.049
GO:0006970	response to osmotic stress	8	0.05

^a^ number of genes associated with the GO term. ^b^ FDR < 0.05 were selected as significant enrichment. ^c^ terms common with BA-repressed terms were highlighted by gray background.

## Data Availability

Data are contained within the article and in the Supplementary Materials (Figures S1–S3, Tables S1–S3).

## Supplementary Materials

The following are available online at https://www.mdpi.com/article/10.3390/life11121404/s1, Figure S1: Benzyladenine (BA) repressed several consecutive steps of JA synthesis, Figure. S2: Effect of kinetin (A) and benzyladenine (B) on gene expressions related to secondary metabolism, Figure. S3: Effect of kinetin (A) and benzyladenine (B) on gene expressions related to secondary metabolism, Table S1: Genes significantly up or downregulated after kinetin or benzyladenine treatments, Table S2: Primers of selected *Arabidopsis* genes used by real-time RT-PCR for validating microarray results, Table S3: Comparison of kinetin and benzyladenine induced *Arabidopsis* gene expression results with previously published cytokinin-responsive gene lists.
