# Supplementary material for: Diverse Effect of Two Cytokinins, Kinetin and Benzyladenine, on Plant Development, Biotic Stress Tolerance, and Gene Expression
Source: Life (Basel). 2021 Dec 15;11(12):1404. doi: 10.3390/life11121404 (PMC8706806; doi:10.3390/life11121404)
Supplement: Supplementary file 1 [file life-11-01404-s001.zip › Table S2.pdf]

Supplementary Table S2

Array ID[a]	Arabidopsis TAIR code[b]	Gene name[c]	Forward primer	Reverse primer	BA microarray[d]	BA RT-PCR[e]	Kin microarray[f]	Kin RT-PCR[g]
A_84_P13657	AT3G29590	At5MAT	5' CGG TTT GTG TGC GTT GGT T 3'	5' TGG TTC TGC TTT CAC GTC GAA 3'	-1.81[h]	-1.83	1.98	1.57
A_84_P808177	AT5G59320	LTP3	5' TAA AAC CAC ACC AGA CCG CC 3'	5' AGA CCA GAA ATG CTC TTC GCA 3'	-4.32	-5.93	1.68	1.23
A_84_P14446	AT2G19190	FRK1	5' AAC GAG AAG CTC CAA GCG AA 3'	5' GGG GTC AAG GTA ACC GAT GG 3'	3.60	2.41	-1.09	-1.71
A_84_P814710	AT1G14870	PCR2	5' CCA AGC CTC ATG CTG AAG GA 3'	5' TAC AAT CTC GGC GAC TTG GC 3'	1.91	1.43	-1.33	-1.03
A_84_P10728	AT2G29110	GLR2.8	5' CCA AAG GGT TAC GCC ATA GAC 3'	5' CTG CAT CCA AAG TCC CGT TAT 3'	1.37	0.32	-1.68	-1.22
A_84_P16678	AT4G25420	GA20OX1	5' CGG TGG TGA ACA GCG AGA GC 3'	5' CTC GGT GGC GTC ACT ACT CTG 3'	2.60	2.51	2.71	1.94
A_84_P167173	AT4G25480	DREB1A	5' ACA GAG GAG TTC GTC GGA GAA 3'	5' CGG CAA CGT CGT GAG C 3'	1.60	1.16	2.02	1.14
A_84_P21915	AT1G20190	EXPA11	5' GCA ATC CGC CGC TTA AAC AT 3'	5' CGG GAA CGA TTC CTC CTC TG 3'	2.11	2.55	1.82	1.71
A_84_P10627	AT2G20880	ERF053	5' CGA GCA CGG CAT TGG CAG T 3'	5' CCA TAG AGG GCT TCC AGG TCC 3'	-2.98	-3.90	-3.28	-4.07
A_84_P19231	AT2G26150	HSFA2	5' TGA GGC AAC AGC AAC ACA GC 3'	5' GGC AAG GAA CGT CAT CAT CTG 3'	-2.33	-3.18	-3.17	-2.69
A_84_P146199	AT5G05440	PYL5	5' GTC CCT GAA CAC GTT GCG ATG 3'	5' CAC AAG AGC CCA CAC GGA CTC 3'	-1.85	-2.02	-2.80	-2.54
A_84_P156615	AT4G12490	AZI3	5' ACC CTA CGC CAG TCA TTC CTC 3'	5' CCG CTT AGC ACA TTC GCA CAT 3'	6.71	10.92		
A_84_P14074	AT5G56970	CKX3	5' GCT GCG TGG CTA TGG ATT GA 3'	5' ATC TGT GGA CCG TAC CGA AAC 3'	5.48	8.95		
A_84_P17268	AT2G14610	PR1	5' GTA GGC GTA GGT CCC ATG CAG 3'	5' CCA GAC AAG TCA CCG CTA CCC 3'	5.53	6.93		
A_84_P610481	AT2G34600	JAZ7	5' TTG GAA CTT CGC CTT CTT ACT TCT T 3'	5' CTG AGA TTC TTG CTT TGG TTG TGA 3'	-5.53	-5.00		
A_84_P544827	AT3G25760	AOC1	5' TCA TCT AAC GGT CCA GGT TCC TC 3'	5' CTT CTT GGG CGT GAA ACT TGT C 3'	-3.98	-3.65		
A_84_P15340	AT2G06050	OPR3	5' ACG TTA CAC AAC CTC GCT ACC AC 3'	5' CAT CAC TCC CTT GCC TTC CA 3'	-3.69	-3.37		
A_84_P11715	AT3G04290	LTL1	5' GGG CTT TCT TTG TCT TTG GTG 3'	5' GGG CAG TGG TGA CGA GGT A 3'			2.88	1.89
A_84_P14899	AT5G10930	CIPK5	5' CGG AAG CGA GGA GGT TGA TT 3'	5' GCT AAC GGC GGA GTG AAG TTT 3'			2.05	1.57
A_84_P21605	AT5G46830	NIG1	5' GAT CCG TCG CCA CCA TTA CT 3'	5' CAG CAT GTA AAC GCT TCG GG 3'			1.65	1.58
A_84_P12477	AT1G79680	WAKL10	5' CGG CAG GCA AGT GTG TCA A 3'	5' CCA CCA ATG AAT ACC AAT GTG CT 3'			-2.68	-2.03
A_84_P555790	AT5G48657		5' TTC CTC CCA CAC CAG AAA CA 3'	5' TAC TTT GCC CTC TAA GAT TGC GTA T 3'			-2.20	-1.37

A_84_P174151	AT2G40330	PYL6	5' TCA GTT TCA GCG TCG TTG GTG 3'	5' CTT CTT GCC GTC GGA GTC CT 3'			-1.98	-0.74

[a] Array probe identifiers of Agilent Arabidopsis 4x44k V4 microarray

[b] Arabidopsis TAIR gene identifiers (www.arabidopsis.org)

[c] common abbreviations of *Arabidopsis* gene names

[d] Gene expression results obtained by Agilent Arabidopsis 4x44k V4 microarray. Benzyladenine (BA) induced transcription levels were compared to water treated controls.

[e] Gene expression results obtained by quantitative RT-PCR. Benzyl adenine (BA) induced transcription levels were normalized to constitutive expressed ubiquitin conjugating enzyme (AT1G14400) and compared to water treated controls.

[f] Gene expression results obtained by Agilent Arabidopsis 4x44k V4 microarray. Kinetin (Kin) induced transcription levels were compared to water treated controls.

[g] Gene expression results obtained by quantitative RT-PCR. Kinetin (Kin) induced transcription levels were normalized to constitutive expressed ubiquitin conjugating enzyme (AT1G14400) and compared to water treated controls.

[h] gene expression in log_2 transformed form. Values are averages of three independent biological replicates. In the case of empty cells the gene expression results did not shown significant changes in microarray experiments.
