# Supplementary material for: Diverse Effect of Two Cytokinins, Kinetin and Benzyladenine, on Plant Development, Biotic Stress Tolerance, and Gene Expression
Source: Life (Basel). 2021 Dec 15;11(12):1404. doi: 10.3390/life11121404 (PMC8706806; doi:10.3390/life11121404)

Figure S1. Benzyladenine (BA) repressed several consecutive steps of JA synthesis. Red and green colors represent up- or down-regulated genes, respectively. The figures were derived from MAPMAN software (Usadel et al. 2005).

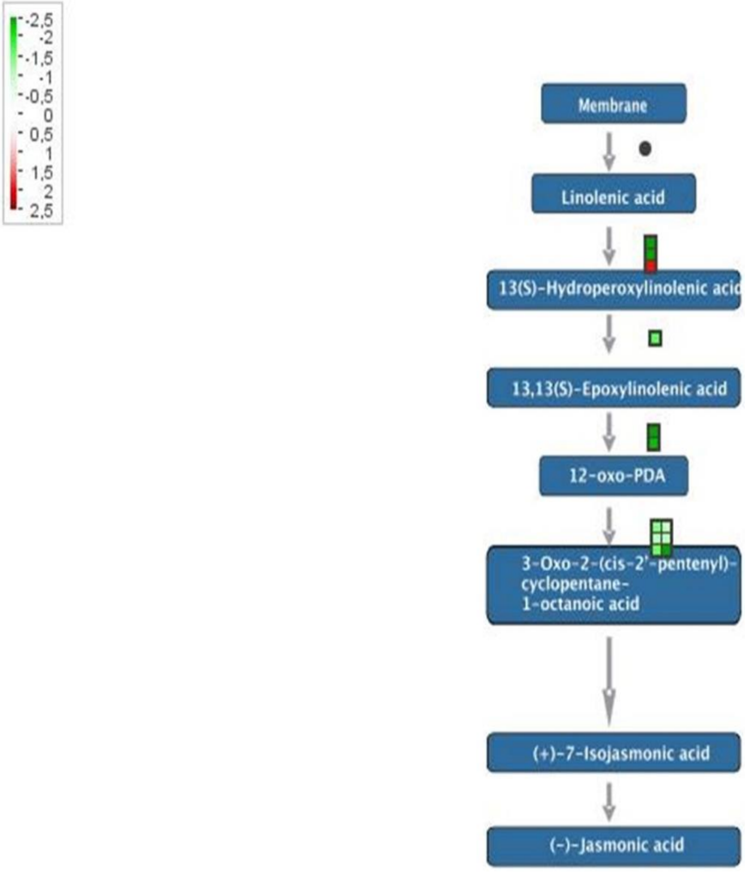

Figure S2. Effect of kinetin (**A**) and benzyladenine (**B**) on gene expressions related to secondary metabolism. Red and green colors represent up- or down-regulated genes, respectively. The figures were derived from MAPMAN software (Usadel et al. 2005).

A

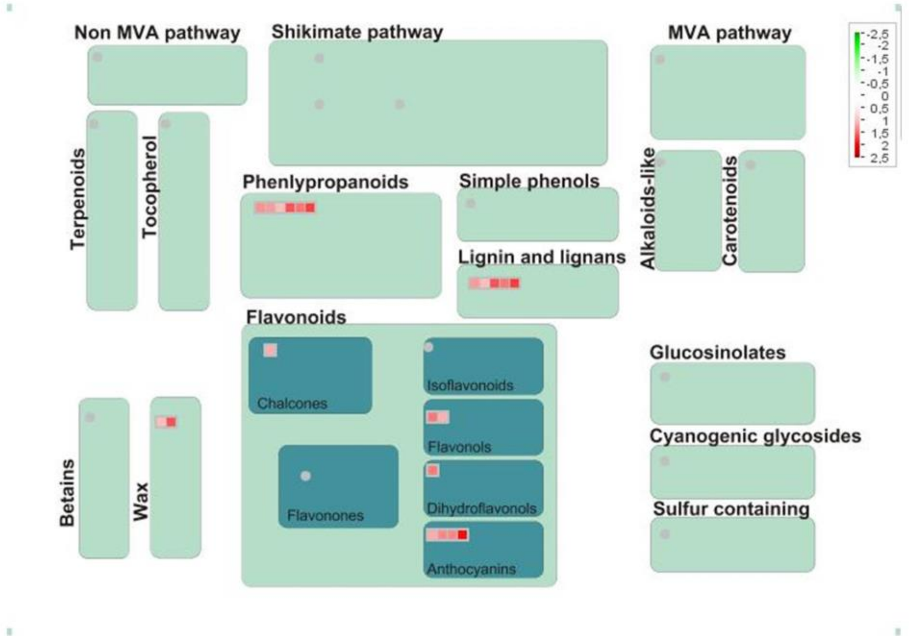

B

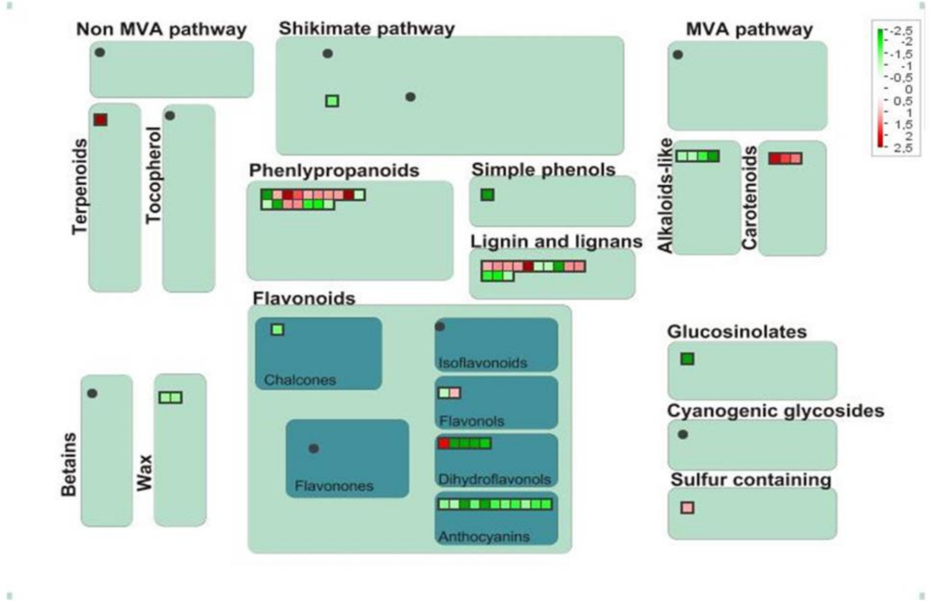

Figure S3. Effect of kinetin (**A**) and benzyladenine (**B**) on gene expressions related to phenylpropanoid/anthocanin synthesis. Red and green colors represent up- or down-regulated genes, respectively. The figures were derived from MAPMAN software (Usadel et al. 2005).

A

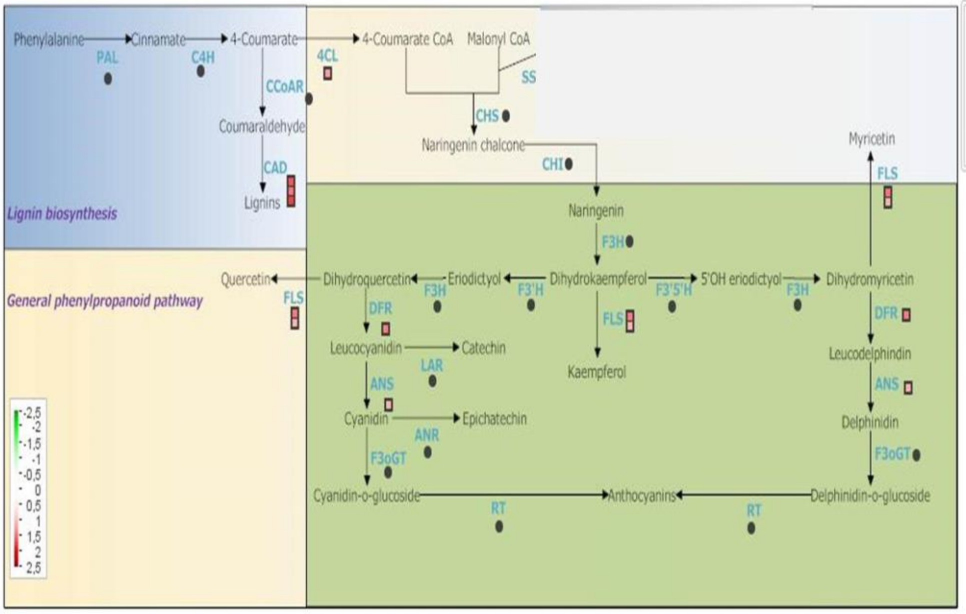

B

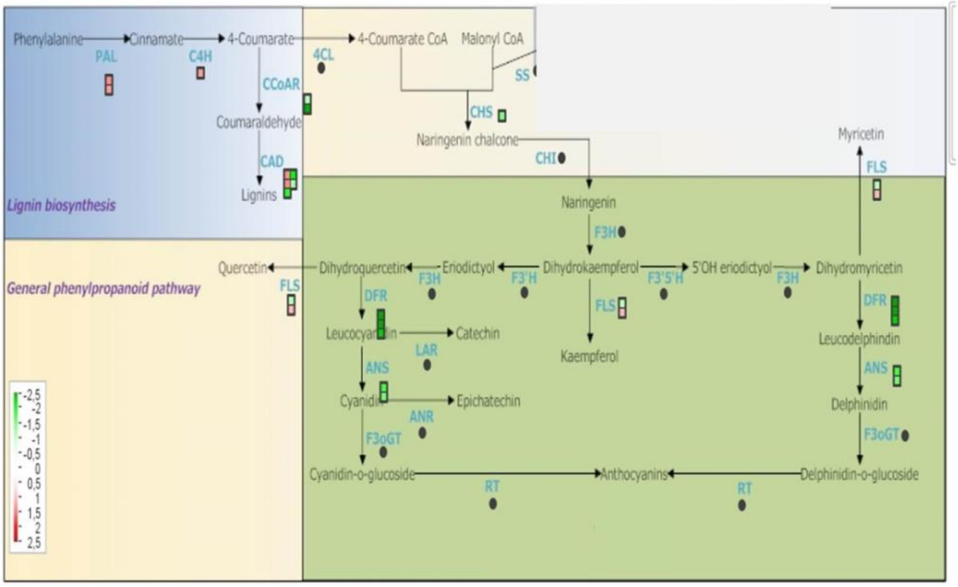

Supplement: Supplementary file 1 [file life-11-01404-s001.zip › Supplementary Figures.pdf]
